# Molecular Testing in Indeterminate Thyroid Nodules: Genomic Landscape, Diagnostic Performance, and Integrated Risk-Stratified Management

**DOI:** 10.3390/cancers18101661

**Published:** 2026-05-21

**Authors:** Sayaka Tanaka, Naomi Kitayama, Kyouko Kawamoto, Tomoko Wakasa, Yanhua Bai, Kennichi Kakudo

**Affiliations:** 1 Department of Legal Medicine, Graduate School of Medicine, Osaka University, Suita 565-0871, Japan; sayka.design@legal.med.osaka-u.ac.jp; 2 Department of Dermatology, Faculty of Medicine, Kindai University, Sakai 590-0197, Japan; naomi.k1226@gmail.com; 3 Department of Pathology, Kindai University Nara Hospital, Ikoma 630-0293, Japan; 230460@med.kindai.ac.jp (K.K.); wakasa@med.kindai.ac.jp (T.W.); 4 Department of Pathology, Peking University Cancer Hospital, Beijing 100142, China; byh0425@sina.com

**Keywords:** thyroid cytology, indeterminate nodule, thyroid cancer, low-risk neoplasm, molecular testing, proteomics, risk stratification, tumor biology

## Abstract

Molecular testing has become an increasingly important adjunct in the evaluation of cytologically indeterminate thyroid nodules. These tests analyze genetic alterations associated with thyroid tumorigenesis, including point mutations, gene fusions, and gene expression profiles, with the aim of refining preoperative risk assessment and reducing unnecessary diagnostic surgery. Despite these advances, the clinical utility of molecular testing remains highly dependent on the context in which results are interpreted. Molecular alterations do not consistently correlate with tumor aggressiveness, and several mutations are observed in both benign and malignant thyroid lesions. In addition, the predictive performance of molecular tests is strongly influenced by the baseline prevalence of malignancy, which varies across clinical settings and is shaped by diagnostic thresholds and patient selection. This commentary summarizes the molecular landscape of thyroid tumors, the diagnostic performance of current molecular testing platforms, and their role in clinical decision-making. Emphasis is placed on the interpretation of molecular findings within a broader diagnostic framework that incorporates cytologic morphology, ultrasound-based risk stratification, and clinical context. A selective, risk-adapted approach to molecular testing may provide the most effective strategy for optimizing patient management while minimizing unnecessary intervention.

## Simple Summary

Molecular testing has become an important adjunct in the evaluation of cytologically indeterminate thyroid nodules by improving preoperative risk stratification and reducing unnecessary surgery. However, molecular alterations do not uniformly predict biological aggressiveness, and several mutations are shared by both benign and malignant lesions. The clinical utility of molecular testing is therefore highly context-dependent and influenced by disease prevalence, diagnostic thresholds, healthcare systems, and local management strategies. This commentary emphasizes that molecular findings should be interpreted within an integrated framework combining cytomorphology, ultrasound risk stratification, clinical assessment, and longitudinal observation when appropriate. A selective, biology-based, and risk-adapted approach may optimize patient management while minimizing overtreatment and preserving oncologic safety.

## 1. Introduction

Thyroid nodules are commonly encountered in clinical practice, and fine-needle aspiration cytology remains the primary diagnostic method for evaluating these lesions. Cytology is highly effective for identifying clearly benign or malignant nodules. However, approximately 20–30% of aspirated nodules fall into indeterminate diagnostic categories within the Bethesda System for Reporting Thyroid Cytopathology [1,2,3].

Indeterminate thyroid nodules present a clinical challenge because cytology alone cannot reliably distinguish benign follicular adenomas from follicular carcinomas. The distinction between these entities requires histologic demonstration of capsular or vascular invasion, which cannot be assessed on cytologic specimens [1]. As a result, many patients with indeterminate cytology have historically undergone diagnostic surgery to establish a definitive diagnosis [1,4,5].

Over the past decade, increasing recognition of the potential harms associated with overdiagnosis and overtreatment of indolent thyroid tumors [6,7,8,9] has stimulated efforts to develop additional tools to refine preoperative risk stratification. Molecular testing has emerged as one such approach. Several molecular diagnostic platforms have been introduced with the goal of improving the evaluation of indeterminate thyroid nodules and reducing unnecessary surgery [10,11,12,13]. However, the clinical value of molecular testing depends not only on the technical performance of the test itself but also on how results are interpreted within the broader clinical context [14,15,16,17].

**Figure 1:**
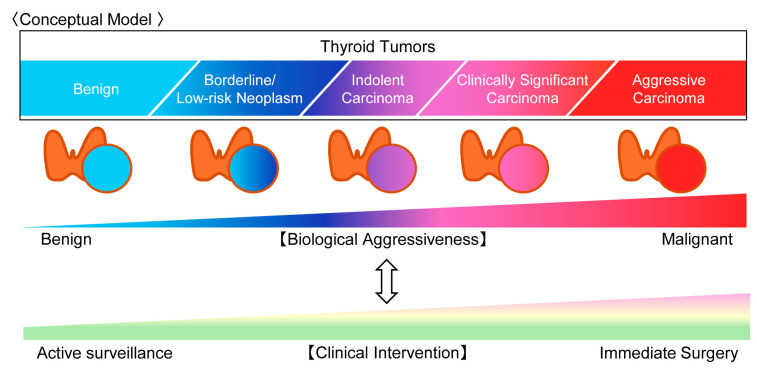
Biological spectrum of thyroid tumor behavior. Thyroid tumors form a continuous biological spectrum ranging from benign lesions to highly aggressive carcinomas. This framework emphasizes graded biological risk rather than binary classification, emphasizing graded risk rather than dichotomous diagnosis. Recognition of this continuum supports risk-adapted management in which the intensity of clinical intervention is aligned with the underlying biological behavior of the tumor.

**Figure 2:**
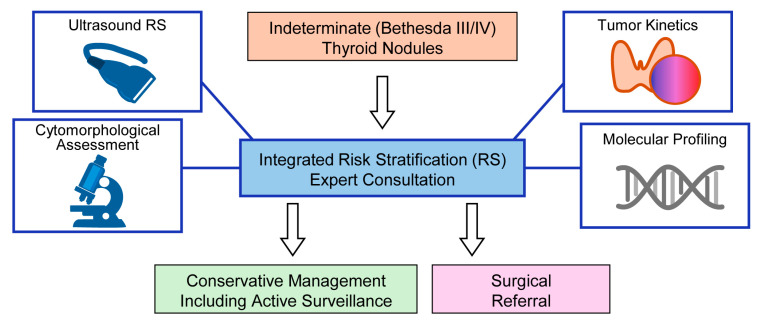
Integrated framework for risk stratification in indeterminate thyroid nodules. Risk assessment in indeterminate thyroid nodules (Bethesda III/IV) is achieved through integration of cytomorphology, ultrasound findings, clinical context, and molecular data. Within this framework, molecular testing functions as a context-dependent modifier that refines, but does not replace, baseline risk estimation. This model defines the role of molecular testing within integrated risk stratification and supports individualized, multidisciplinary decision-making.

This commentary examines the molecular landscape of thyroid tumors and evaluates the role of molecular testing in the management of indeterminate thyroid nodules. Particular emphasis is placed on the integration of molecular findings with cytologic morphology and clinical risk stratification. This conceptual framework is illustrated in Figure 1 and Figure 2.

## 2. Molecular Landscape of Thyroid Tumors

The molecular landscape of thyroid cancer has been increasingly clarified through large-scale genomic studies, particularly The Cancer Genome Atlas analysis of papillary thyroid carcinoma [11]. These studies demonstrated that most thyroid tumors harbor mutually exclusive driver alterations that activate key oncogenic signaling pathways, particularly the mitogen-activated protein kinase (MAPK) pathway. Integrated genomic analyses have identified two major molecular subtypes of papillary thyroid carcinoma: *BRAF*-like and *RAS*-like tumors. *BRAF*-like tumors typically harbor the *BRAF V600E* mutation and display classical papillary thyroid carcinoma morphology with papillary architecture and characteristic nuclear features. These tumors often show reduced expression of thyroid differentiation genes and may exhibit more aggressive biological behavior in certain clinical contexts [11]. In contrast, *RAS*-like tumors include follicular adenomas, follicular carcinomas, and follicular-patterned variants of papillary thyroid carcinoma [17,18,19,20]. These tumors generally maintain follicular cell differentiation and exhibit follicular architectural patterns. Importantly, *RAS* mutations occur in both benign and malignant follicular-patterned lesions, limiting their specificity for malignancy in cytologic specimens [21,22,23,24]. Recent studies have also suggested that selected *RAS*-mutated indeterminate nodules may remain stable during active surveillance, indicating that molecular positivity alone does not necessarily mandate immediate surgical intervention [24,25].

Additional recurrent genetic alterations include gene fusions involving *RET*, *NTRK*, *ALK*, and *BRAF*, as well as mutations affecting the telomerase reverse transcriptase (*TERT*) promoter, phosphatidylinositol 3-kinase (PI3K)–AKT pathway, and *TP53*. Many of these alterations activate the MAPK pathway or the PI3K–AKT signaling pathway and contribute to tumor initiation or progression. The identification of certain gene fusions has also gained clinical relevance, as some of these alterations may represent potential targets for kinase inhibitor therapy, particularly in advanced disease settings [18,26]. Among the molecular alterations identified in thyroid cancer, *TERT* promoter mutations have emerged as important markers associated with aggressive tumor behavior. These mutations occur in approximately 5–15% of papillary thyroid carcinomas and are more frequently observed in advanced or poorly differentiated tumors [27,28,29,30]. Several studies have demonstrated that the coexistence of *BRAF V600E* and *TERT* promoter mutations is associated with markedly increased risks of recurrence, distant metastasis, and disease-specific mortality [27,28,29,30].

Similar synergistic interactions have also been described between *RAS* mutations and *TERT* promoter mutations, particularly in follicular-patterned thyroid cancers [21,22,29]. In more advanced thyroid cancers, including poorly differentiated and anaplastic carcinomas, additional mutations involving *TP53, EIF1AX*, and *CTNNB1* are frequently observed and are thought to contribute to tumor dedifferentiation and aggressive clinical behavior [21,28,29,30,31].

Taken together, these findings illustrate that thyroid tumorigenesis is driven by a spectrum of genetic alterations involving multiple signaling pathways [20,21,22,30]. Understanding this molecular landscape provides an essential biological framework for the development and clinical application of molecular diagnostic testing in the evaluation of indeterminate thyroid nodules. These advances in tumor genomics have directly stimulated the development of molecular diagnostic tests designed to improve the evaluation of cytologically indeterminate thyroid nodules [32,33,34,35,36].

## 3. Molecular Testing Platforms (Table 1)

Several molecular testing platforms have been developed to assist in the evaluation of indeterminate thyroid nodules. These tests aim to improve preoperative risk stratification by analyzing molecular alterations associated with thyroid tumorigenesis [32,33,34,35,36,37].

**Table 1:** Major molecular testing platforms for indeterminate thyroid nodules.

Molecular Test	Technology	Major Targets	Diagnostic Role	Key Strengths	Limitations
**Afirma Gene Expression Classifier** (GEC/GSC)	RNA expression profiling	Whole-transcriptome expression signatures	Primarily **rule-out test**	High negative predictive value; helps avoid diagnostic surgery in benign nodules	Limited specificity; does not identify specific oncogenic drivers
**ThyroSeq (v3)**	Next-generation sequencing (DNA/RNA panel)	*BRAF*, *RAS*, *TERT* promoter, *RET*, *NTRK*, *ALK* fusions, multiple additional genes	**Rule-in and rule-out** depending on detected alterations	Comprehensive genomic profiling; identifies actionable mutations	Some mutations occur in benign lesions (e.g., *RAS*); interpretation requires clinical context
**ThyGenX + ThyraMIR**	Targeted mutation panel + microRNA classifier	*BRAF*, *RAS*, *RET*/*PTC*, *PAX8*–*PPARG*; microRNA signatures	Combined **rule-in and rule-out** approach	Integrates mutation analysis with microRNA risk stratification	Performance varies across cohorts; interpretation may depend on disease prevalence
**RosettaGX Reveal**	microRNA expression profiling	microRNA expression signatures	Primarily **rule-out test**	Can be performed on cytology smear slides	Lower availability; variable validation across institutions

Molecular testing platforms provide complementary approaches to risk stratification in nodules with indeterminate cytology. Gene expression classifiers primarily function as rule-out tests with high negative predictive value, whereas next-generation sequencing panels may provide both rule-in and rule-out information depending on the detected alterations. The clinical utility of these tests is context-dependent and relies on integration with cytomorphology, ultrasound findings, and clinical risk assessment.

### 3.1. Gene Expression Classifiers

Gene expression classifier tests analyze RNA expression patterns to determine whether a thyroid nodule is likely benign or malignant. Representative platforms include the Afirma Gene Expression Classifier and the Afirma Gene Sequencing Classifier, which evaluate transcriptomic signatures derived from thyroid nodules with indeterminate cytology. These assays are generally designed as rule-out tests, providing a high negative predictive value [32,34,35]. A benign result from a gene expression classifier may therefore support conservative management and help avoid diagnostic surgery in selected patients with indeterminate cytology.

### 3.2. Next-Generation Sequencing Panels

Next-generation sequencing panels analyze a broad range of genetic alterations, including point mutations and gene fusions. The ThyroSeq assay is a representative next-generation sequencing-based platform designed to detect multiple driver mutations and gene fusions associated with thyroid tumorigenesis [13,36,37,38,39]. These assays may function as rule-in or rule-out tools depending on the detected genetic alterations. In addition to large multigene panels widely used in North America, several centers in Europe and China employ more limited molecular testing strategies [15,37,38,39,40]. These approaches often involve targeted gene panels that include common driver alterations such as *BRAF*, *RAS*, *RET/PTC*, and *PAX8–PPARG* [15,39,40]. In some institutions, particularly in regions with a high prevalence of papillary thyroid carcinoma, single-gene testing for *BRAF V600E* is used as a cost-effective adjunct to cytology [39,41]. While these assays provide valuable genomic information, their clinical interpretation requires integration with cytologic, imaging, and clinical findings. Reported diagnostic performance also varies according to baseline risk of malignancy, referral patterns, and patient selection. Consequently, positive and negative predictive values observed in one healthcare setting may not be directly generalizable to other clinical environments with different disease prevalence and diagnostic pathways [15,16,42,43]. Importantly, the clinical utility of molecular testing varies across healthcare systems depending on disease prevalence, diagnostic pathways, and resource availability [43,44].

## 4. Diagnostic Performance of Molecular Testing

The diagnostic performance of molecular tests is typically evaluated using measures such as sensitivity, specificity, positive predictive value, and negative predictive value. Reported performance varies substantially among studies because of differences in patient populations, prevalence of malignancy, and study design [15,32,44]. In general, rule-out tests demonstrate high negative predictive value, whereas rule-in tests show higher positive predictive value for specific mutations. Another important limitation is that molecular alterations do not always correlate with tumor aggressiveness. Some tumors harbor oncogenic mutations such as *BRAF V600E* yet behave in an indolent fashion [45]. Furthermore, several genetic alterations—including *RAS* mutations—are detected in both benign and malignant thyroid lesions, limiting their specificity for malignancy [21,22,23,24,25]. Conventional estimates of risk of malignancy in indeterminate cytology may also be affected by selection bias, as they are often derived from surgically resected nodules. Because surgically treated cohorts are enriched for clinically suspicious nodules, reported diagnostic accuracy metrics may overestimate real-world performance when applied to broader populations of indeterminate nodules. To address this limitation, molecular-derived estimates of malignancy risk have been proposed based on probability outputs of molecular classifiers, which may better reflect risk across the broader population of indeterminate nodules [14,46].

Recent advances in molecular profiling technologies have expanded the characterization of thyroid tumors beyond genomic alterations alone. Proteomic profiling provides complementary information by reflecting functional consequences of genomic changes and interactions within the tumor microenvironment. Emerging evidence suggests that integrating genomic, proteomic, and clinical data may improve diagnostic performance in the evaluation of indeterminate thyroid nodules [47,48,49,50]. These integrative approaches may also have potential value in selected complex clinical settings, including large or biologically heterogeneous thyroid tumors [51]. However, these emerging multi-omics approaches still face important challenges, including limited external validation, interplatform reproducibility, cost, and lack of standardization across institutions. In addition, the clinical availability and real-world feasibility of advanced proteomic and multi-omics testing remain variable across healthcare systems.

The economic implications of molecular testing have been widely discussed; however, cost-effectiveness is highly dependent on healthcare system structure and local clinical practice patterns and is beyond the scope of this review.

## 5. Clinical Implications

Molecular testing has expanded the range of diagnostic tools available for evaluating indeterminate thyroid nodules. In clinical practice, molecular findings are interpreted in conjunction with cytologic morphology, ultrasound features, and patient-specific clinical factors to guide management decisions. These tests may be particularly valuable when cytologic and imaging assessments are discordant or inconclusive. The identification of oncogenic fusions has gained increasing clinical relevance, as several of these alterations may have therapeutic implications, particularly in advanced disease settings [26,38,42]. However, the presence of a molecular alteration alone does not determine clinical management, as biological behavior varies widely even among tumors sharing similar genetic profiles. When cytologic morphology and imaging findings consistently indicate either low-risk or high-risk disease, molecular testing is unlikely to provide substantial additional value. In such cases, management decisions can often be guided by established clinical risk stratification systems. Therefore, the optimal role of molecular testing is within a selective, risk-adapted diagnostic framework that incorporates cytologic interpretation, imaging findings, and clinical context. Within this framework, molecular testing functions as an adjunct to refine risk estimation rather than as a standalone determinant of clinical decision-making (Figure 2). A key role of molecular testing within this framework is to refine risk estimation when conventional diagnostic modalities yield indeterminate or conflicting results. Rather than replacing cytologic or imaging assessment, molecular findings modify the estimated probability of malignancy within a Bayesian framework. In this integrated model, cytomorphology and ultrasound findings establish the baseline risk, while molecular testing provides incremental information that may shift clinical decision-making toward either surveillance or surgical intervention [32,33,43,44]. This integrative approach reflects a shift from binary diagnosis toward probabilistic, biology-based risk stratification. Accordingly, the value of molecular testing lies not in its independent diagnostic accuracy, but in its ability to recalibrate risk within a multidimensional assessment of tumor biology (Figure 2).

## 6. Diagnostic Thresholds, Indeterminate Category Utilization, and System Behavior

Diagnostic thresholds influence the distribution of cytologic categories and the proportion of nodules referred for surgery [17,52,53,54,55,56]. As a result, the pretest probability of malignancy varies substantially across institutions and healthcare systems, directly affecting the predictive performance and clinical impact of molecular testing. These variations often reflect differences in diagnostic pathways, category utilization, and patient selection rather than intrinsic differences in tumor biology.

Within the Bethesda System for Reporting Thyroid Cytopathology, indeterminate categories such as atypia of undetermined significance (AUS; Bethesda III) and follicular neoplasm (FN; Bethesda IV) are intended to represent a limited subset of cases in which cytologic findings are insufficient for definitive classification [1]. However, substantial interinstitutional and international variation in the utilization of these categories has been reported [17,54,55]. These differences are closely related to diagnostic thresholds applied during cytologic interpretation and directly influence the number of patients entering molecular testing and surgical pathways.

Several Asian practice settings have reported relatively low frequencies of FN diagnoses, often around 3%, compared with higher rates in many Western series [56,57]. In settings where FN utilization remains around 3%, the proportion of surgically resected nodules with malignancy may exceed 60–70%, whereas broader FN utilization lowers baseline ROM and alters downstream molecular test performance. These findings illustrate how category utilization influences not only surgical selection but also the apparent diagnostic accuracy and clinical utility of molecular assays.

Broader use of indeterminate categories increases the number of nodules subjected to molecular testing and diagnostic surgery, including lesions with lower baseline risk. In contrast, stricter diagnostic thresholds may reduce downstream intervention by resolving diagnostic uncertainty earlier in the evaluation process. Accordingly, the clinical performance of molecular testing should be understood as context-dependent and shaped by upstream diagnostic behavior as well as pretest malignancy prevalence.

Importantly, molecular testing has also been proposed as a potential quality metric in thyroid cytopathology practice. Molecular-derived risk estimates and test utilization patterns may provide indirect feedback regarding how frequently indeterminate categories are assigned and how effectively they stratify biological risk [14,58].

Taken together, these observations indicate that optimization of thyroid nodule management requires consideration of both upstream diagnostic thresholds and downstream molecular interpretation. Molecular testing is most appropriately viewed as one component of an integrated and system-dependent risk stratification framework rather than as an isolated diagnostic solution.

## 7. System-Level Interpretation of Molecular Testing

The clinical utility of molecular testing depends not only on the analytical performance of the assay itself, but also on how testing is incorporated into broader diagnostic and management pathways. Molecular results are interpreted within healthcare systems that differ substantially in cytologic thresholds, ultrasound utilization, surgical referral patterns, reimbursement structures, and tolerance for active surveillance [16,43,44,56,57]. Consequently, the same molecular alteration or classifier result may lead to different clinical decisions across practice environments.

In settings with highly structured cytopathology and ultrasound-based risk stratification, molecular testing is often used selectively in cases with discordant or borderline findings. Under these conditions, molecular assays primarily function as adjunctive tools that refine pretest risk established by cytomorphology and imaging. In contrast, in healthcare environments with broader utilization of indeterminate cytologic categories or lower thresholds for surgical referral, molecular testing may assume a more central role in downstream decision-making. These differences influence not only test utilization rates but also the observed predictive performance of molecular platforms, including positive predictive value and negative predictive value [14,15,16,17,43,44].

Differences between Asian and Western thyroid nodule practice illustrate the importance of healthcare context in interpreting molecular diagnostics. Several Asian practice settings have reported relatively low frequencies of indeterminate diagnoses and lower surgical resection rates, while maintaining relatively high malignancy rates among resected nodules [16,55,56]. In such systems, risk stratification is often achieved through integrated interpretation of cytomorphology, ultrasound findings, and longitudinal observation, with molecular testing used more selectively. In contrast, molecular testing is more extensively incorporated into routine management algorithms in some Western practice settings, particularly where indeterminate categories are more broadly utilized and diagnostic surgery has historically played a larger role in thyroid nodule management [15,16,43,44].

These observations indicate that molecular testing does not function as a universally interchangeable diagnostic solution. Rather, its clinical role is shaped by the surrounding diagnostic ecosystem, including disease prevalence, patient selection, local management philosophy, and healthcare resource allocation. Accordingly, interpretation of molecular findings should remain integrated, context-dependent, and aligned with multidisciplinary clinical decision-making rather than viewed as an isolated determinant of management (Figure 2).

## 8. Detection–Intervention Mismatch

**Figure 3:**
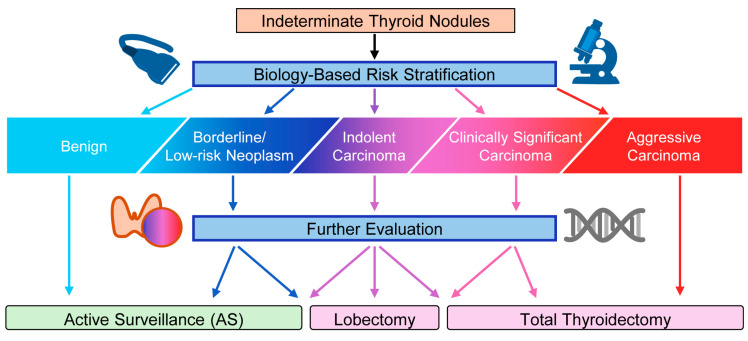
Biology-aligned management pathways in thyroid nodule care. Clinical management of thyroid nodules is aligned with biological risk across the disease spectrum. Low-risk lesions are typically managed with observation or active surveillance, whereas high-risk tumors require surgical intervention; intermediate lesions are guided by integrated, probabilistic risk assessment incorporating morphology, imaging, molecular findings, and tumor kinetics. This framework aligns management intensity with biological risk, aiming to minimize unnecessary intervention while maintaining oncologic safety.

A fundamental challenge in modern thyroid oncology is the potential mismatch between advances in disease detection and the intensity of subsequent clinical intervention. Improvements in imaging, cytologic interpretation, and molecular diagnostics have markedly increased the detection of small thyroid nodules and early-stage cancers [6,7,8,59,60]. However, many of these lesions—particularly small intrathyroidal papillary thyroid carcinomas—exhibit indolent biological behavior and are unlikely to cause clinically significant harm during a patient’s lifetime [61,62,63]. When detection identifies lesions with limited malignant potential, the clinical benefit of intervention becomes less certain. In such cases, aggressive diagnostic or therapeutic strategies may expose patients to surgical risks, lifelong thyroid hormone replacement, and the psychological burden of a cancer diagnosis without meaningful improvement in survival [4,5,20,61,62]. This imbalance highlights a detection–intervention mismatch, in which the capacity to detect disease exceeds the ability to discriminate biologically significant tumors from indolent lesions. Addressing this mismatch requires diagnostic approaches that refine risk assessment rather than simply increase detection sensitivity. Integration of cytologic morphology, molecular testing, imaging findings, and clinical context may help align the intensity of intervention with tumor biology (Figure 3).

A key limitation of current molecular biomarkers is that they predict future tumor behavior based on a single time-point observation. Genomic and proteomic profiles capture the biological state of a tumor at the moment of sampling but do not incorporate the temporal dynamics of tumor evolution. As a result, even advanced molecular classifiers cannot reliably predict outcomes in all patients and therefore categorize nodules in probabilistic terms. In contrast, longitudinal observation provides direct information on tumor kinetics, including changes in tumor size and progression over time [45,61,63,64]. Incorporating time-dependent data through active surveillance or structured follow-up may therefore provide a critical complementary dimension for predicting tumor behavior and guiding management decisions. Tumor kinetics derived from serial observation may represent one of the most informative indicators of biological aggressiveness in thyroid nodules. This mismatch underscores the need for diagnostic strategies that not only improve detection but also enhance biological risk discrimination. Integration of molecular data with morphology, imaging, and longitudinal observation may help bridge this gap by aligning intervention with tumor behavior (Figure 3).

## 9. Conclusions

Molecular testing has expanded the diagnostic tools available for evaluating cytologically indeterminate thyroid nodules by providing additional information on tumor biology and malignancy risk. However, molecular alterations should not be interpreted as deterministic indicators of clinical behavior, as many genetic changes occur across lesions with widely variable biological potential. The clinical value of molecular testing depends on integrated interpretation with cytomorphology, ultrasound findings, clinical context, and, when appropriate, longitudinal tumor kinetics. Within this framework, molecular testing functions as a context-dependent modifier of baseline risk rather than a standalone determinant of management decisions. Its performance and clinical impact are strongly influenced by disease prevalence, diagnostic thresholds, and healthcare systems. A biology-based, risk-adapted approach may help reduce unnecessary intervention while maintaining oncologic safety. Future progress will likely depend on improved integration of molecular, imaging, and clinical data, including emerging multimodal and artificial intelligence-assisted risk stratification models.

## Data Availability

No research data available.
